# A review on renewable energy: Conversion and utilization of biomass

**DOI:** 10.1002/smo.20240019

**Published:** 2024-10-10

**Authors:** Xinping Yang, Yongjia Zhang, Peiliang Sun, Chong Peng

**Affiliations:** ^1^ State Key Laboratory of Fine Chemicals School of Chemical Engineering Dalian University of Technology Dalian China; ^2^ Mechanical Engineering International Institute for Carbon Neutral Energy Research Kyushu University Fukuoka Japan

**Keywords:** biomass conversion, biomass value‐added, lignocellulose, lignocellulose pretreatment, platform chemicals

## Abstract

The significant increase in demand for fuels and chemicals driven by global economic expansion has exacerbated concerns over fossil fuel consumption and environmental pollution. To achieve sustainable production of fuels and chemicals, biomass resources provide a rich repository for carbon‐neutral, green renewable energy, and organic carbon. This paper reviews the transformation and utilization of lignocellulosic biomass and its derivatives, emphasizing their valorization into high‐quality chemicals and biofuels. The advantages and disadvantages of various pretreatment methods are discussed based on the composition of lignocellulose. Furthermore, the methods and pathways for the valorization and conversion of cellulose, hemicellulose, and lignin are detailed according to the unique functional groups of different lignocellulosic platform molecules. However, the complex and resilient structure of biomass presents challenges for the disassembly and utilization of single components, and achieving high yields and selectivity for target products remains difficult. In conclusion, this paper comprehensively reviews the various types and pretreatment technologies of lignocellulose, focusing on the methods and pathways for the valorization of lignocellulosic biomass and its derivatives, thereby providing clear guidance and insights for optimizing lignocellulose utilization in the future.

## Graphical abstract

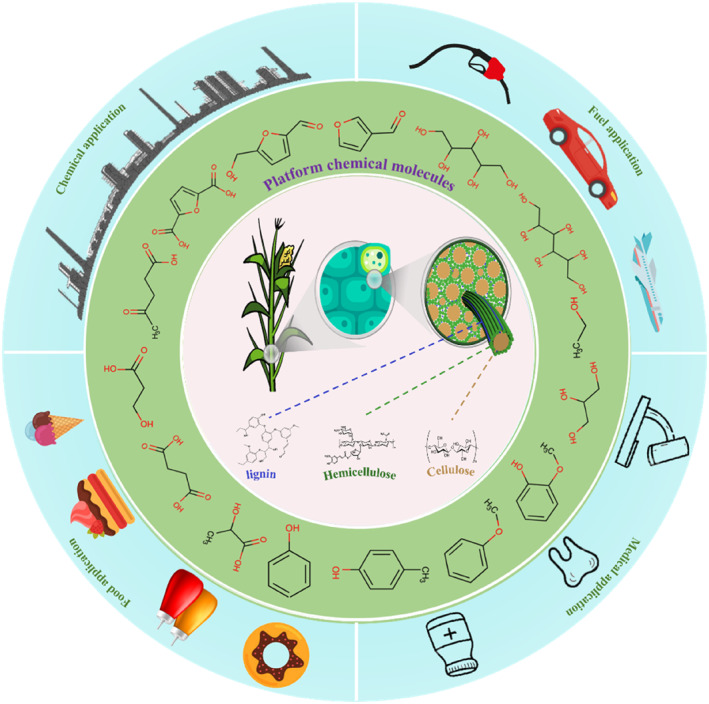

## 1 INTRODUCTION

With the swelling of the Earth's population and the industrialization of developing countries, humanity's demand for energy has reached unprecedented levels.^[1]^ Currently, the widely used energy and chemicals are primarily derived from fossil fuels such as coal, oil, and natural gas. It is estimated that since the advent of commercial oil drilling in the 1850s, we have extracted over 135 billion tons of crude oil to power our vehicles, fuel our power plants, and heat our homes. This number is increasing daily, and over the past 200 years, global fossil fuel consumption has grown by more than 1300 times. However, fossil fuels take millions of years to form, and the rate of consumption far exceeds the rate of formation. Additionally, the consumption of fossil fuels brings numerous environmental issues, such as soil pollution, extreme weather, and the greenhouse effect.^[2]^ Therefore, addressing the contradiction between the growing development needs and environmental protection has become one of the major challenges facing humanity.

Energy is a crucial strategic resource for national economies. To achieve sustainable development, countries are vigorously researching and utilizing renewable resources such as solar energy, wind energy, hydropower, and biomass to reduce environmental damage and reliance on fossil fuels.^[3]^ Among these renewable energy sources, biomass is the only resource capable of continuously producing liquid fuels and chemical raw materials for transportation, making it the fourth‐largest energy source after crude oil, coal, and natural gas.^[4]^ Biomass resources include forest residues (hardwood, softwood, and grasses), agricultural waste (straw, husks, and stalks), aquatic biomass (algae), and various types of organic waste, which are widely distributed around the world. These resources have the advantages of vast reserves, environmental friendliness, and broad application scope.[^5^, ^6^] Additionally, biomass mainly consists of lignocellulose, lipids, and a small portion of proteins, with lignocellulose accounting for over 90% of all plant biomass. Lignocellulose is non‐edible and does not compete with food supplies.[^7^, ^8^] Lignocellulose contains substantial amounts of carbon, hydrogen, and oxygen, providing a wealth of chemical bonds and functional groups that facilitate various chemical reactions. This is significant for improving the current energy structure, alleviating the over‐reliance on fossil fuels, and reducing environmental pollution.

The high‐value conversion of biomass has significant scientific implications for addressing the issue of energy shortages.^[9]^ One of the key technologies lies in the efficient and selective activation and cleavage of C‐O bonds and the selective hydrogenation of C=O bonds. By utilizing different substrates through hydrodeoxygenation (HDO) technology, a variety of chemicals and biofuels can be produced. For example, after pretreatment and HDO of lignocellulose, various hydrocarbon compounds can be generated, which can be used as fuels, solvents, or industrial raw materials. Specifically, cellulose and hemicellulose can be converted into sugars, which can then undergo further chemical reactions to produce ethanol or other liquid fuels; lignin can be treated via HDO to obtain phenolic or other aromatic compounds. Despite significant progress in biomass conversion technology at the laboratory level, existing systems still face several challenges. Firstly, the complex structure of biomass makes the efficient separation and conversion processes difficult. Secondly, HDO reactions typically require high temperature and pressure conditions, resulting in high energy consumption and catalyst deactivation. Additionally, current technologies struggle to achieve high selectivity and yield of target products, leading to low product purity and necessitating complex and costly further separation and purification processes.

**FIGURE 1:**
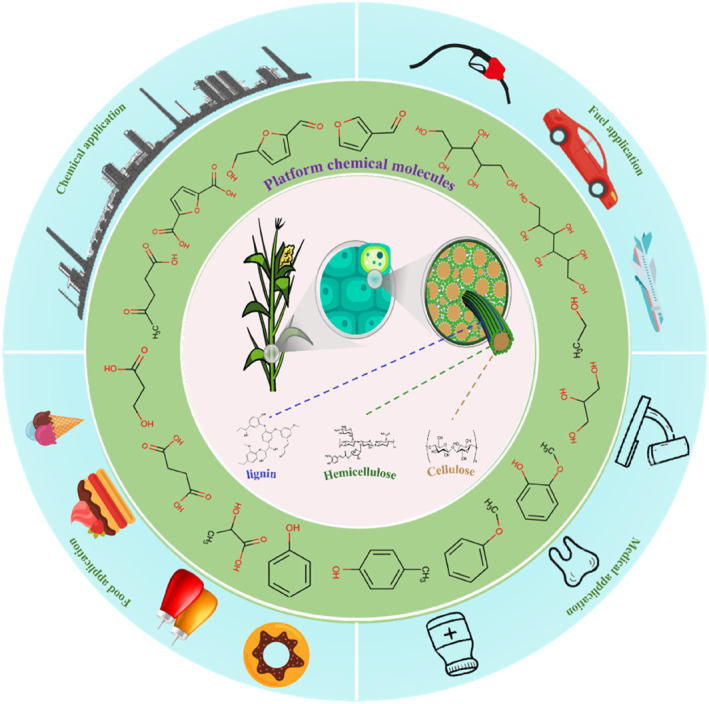
Schematic diagram of lignocellulose value addition.

In recent years, many reviews have primarily focused on the conversion of biomass into biofuels or its value‐added through various methods, such as pretreatment methods, conversion techniques, and types of catalysts. Therefore, this paper comprehensively summarizes the latest progress in the conversion of lignocellulose into high‐value chemicals and fuels. While briefly introducing the structure of biomass, it discusses the advantages and disadvantages of different pretreatment methods and further explores the main pathways and methods for the value‐added of cellulose/hemicellulose and lignin (Figure 1). In the future, the development of biomass conversion technology will focus on the design and development of efficient catalysts, particularly those with high activity, selectivity, and stability, as well as the optimization of biocatalysts. In terms of process integration and optimization, coupling different conversion technologies with intelligent control can enhance overall efficiency and economic viability. The advancement of biomass value‐added technologies will promote the efficient and sustainable utilization of biomass resources, providing a solid technical foundation for the development of a green economy.

## 2 COMPOSITION OF LIGNOCELLULOSE

**FIGURE 2:**
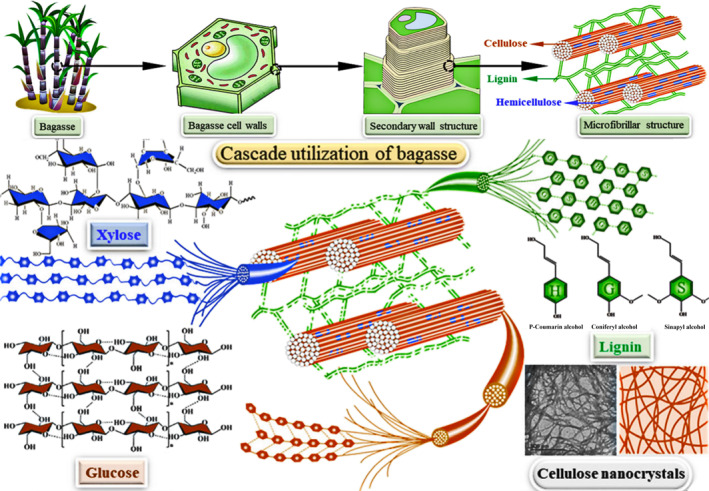
Schematic diagram of the lignocellulose composite structure inside the plant cell wall. Reproduced with permission.^[12]^ Copyright 2021, Springer.

The plant cell wall consists of primary and secondary walls, with pectin being the main component of the middle lamella between adjacent cells. The primary wall primarily comprises cellulose, hemicellulose, and pectin, which are relatively thin, soft in texture, and exhibit good plasticity. In contrast, the secondary wall is generally thicker and harder due to the presence of lignin, enhancing the mechanical strength of the cell wall.^[10]^ Lignocellulose is primarily a carbohydrate polymer, including cellulose, hemicellulose, and lignin, predominantly distributed in the secondary walls of plant cell walls, making it a highly porous biomaterial.^[11]^ Cellulose in the secondary wall of plant cells exhibits a specific arrangement as illustrated in Figure 2.^[12]^ Cellulose serves as the structural framework of the cell wall, with a dense and orderly arrangement of carbon chains, exhibiting high parallelism between chains. Hemicellulose, as sugar chains, intertwines between cellulose molecules, and lignin, a complex three‐dimensional structure of polyphenols, expands into a network structure, enveloping cellulose and hemicellulose within it. The composition of lignocellulose varies depending on factors such as origin, growth stage, storage environment, cultivation conditions, soil minerals, fertilizers, plant species, and biological types, leading to changes in the content of inorganic and organic components. Table 1 shows the content of cellulose, hemicellulose, and lignin in different agricultural residues.^[13]^ Although the composition of lignocellulose differs among different biomasses, cellulose, hemicellulose, and lignin constitute the majority of all lignocellulosic biomasses. The typical composition of lignocellulose is cellulose (35–55 wt%), hemicellulose (20–40 wt%), and lignin (10–25 wt%).^[14]^

**TABLE 1:** Contents of cellulose, hemicellulose and lignin in various common agricultural wastes.

Agricultural waste	Cellulose (%)	Hemicellulose (%)	Lignin (%)
Corn stalks	35.0–39.6	16.8–35.0	7.0–18.4
Rice straw	29.2–34.7	23.0–25.9	17.0–19.0
Wheat straw	35.0–39.0	23.0–30.0	12.0–16.0
Sorghum straw	32.0–35.0	24.0–27.0	15.0–21.0
Chaff	28.7–35.6	12.0–29.3	15.4–20.0
Corn cob	33.7–41.2	31.9–36.0	6.1–15.9
Bagasse	25.0–45.0	28.0–32.0	15.0–25.0

### 2.1 Cellulose

Cellulose, a macromolecular polysaccharide, comprises D‐glucose units linked by β‐1,4‐glycosidic bonds with a molecular weight ranging from approximately 50,000–2,500,000, corresponding to 300–15,000 glucose units.^[15]^ Structurally, the cellulose molecule chain is a linear macromolecule devoid of long side chains, forming a linear polymer. Characterized by an orderly and organized molecular arrangement, cellulose exhibits a high degree of parallelism between its carbon chains, resulting in an extendable flat double‐helix crystal structure.^[16]^ The presence of numerous hydroxyl groups within the sugar units facilitates the formation of hydrogen bonds in the cellulose molecule chain. These hydrogen bonds, being directional in nature, promote the oriented arrangement of cellulose molecules and aid in cellulose crystallization. However, internal hydrogen bonds tend to distribute charges relatively evenly within the cellulose molecule, thereby reducing the directional forces between molecules and impeding crystallization. Cellulose manifests as a partially crystalline polymer, and its crystallinity, denoting the ratio of ordered regions to disordered regions within the polymer, significantly influences material strength and stiffness. Generally, higher relative crystallinity imparts greater mechanical toughness to cellulose, thereby slowing down the conversion process.^[17]^

### 2.2 Hemicellulose

Hemicellulose, a heteropolymer polysaccharide, coexists with cellulose in the majority of plant cell walls, comprising branched polymers of diverse sugar units, with a polymerization degree ranging from 500 to 3000.^[18]^ It encompasses various polysaccharides such as xylan, polygalactose‐glucomannose, polyarabinose‐galactose, glucomannan, and polydextrose‐mannose.^[13]^ These heteropolymers consist of different monosaccharides, predominantly pentoses (xylose, arabinose), hexoses (glucose, mannose, galactose), minor sugars (rhamnose and fructose), uronic acids, and acetyl groups, interconnected via hydrogen bonds and covalent bonds. Unlike cellulose, hemicellulose adopts a random non‐crystalline structure with lower stability, engaging in interactions with crystalline cellulose and amorphous lignin through hydrogen bonding and other non‐covalent attractions. Due to their hydrophilic nature, hemicelluloses are more readily accessible to pretreatment chemicals and biocatalysts, rendering them more susceptible to depolymerization and conversion into small molecule compounds compared to cellulose and lignin.^[19]^

### 2.3 Lignin

**FIGURE 3:**
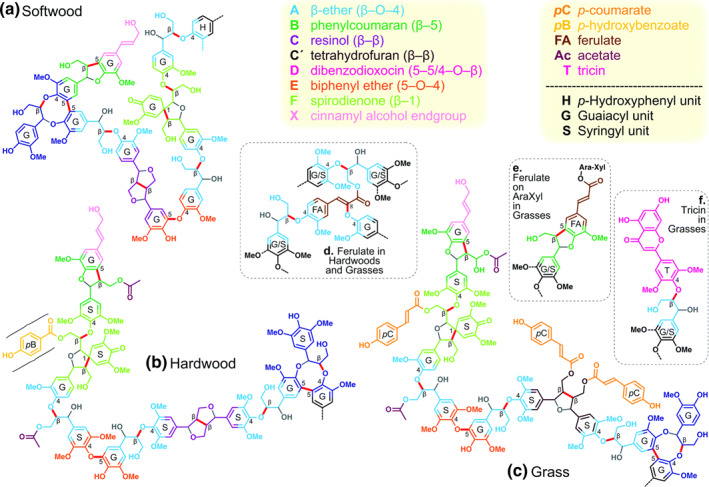
Chemical structure of different types of lignin. Reproduced with permission.^[21]^ Copyright 2021, Royal Society of Chemistry.

Lignin, a complex organic polymer, plays a pivotal role as a structural component in the supportive tissues of certain algae and vascular plants. It provides these tissues with flexibility, resilience, efficient water transport, and resistance to pests and pathogens within the xylem and phloem cells.^[15]^ Structurally, lignin constitutes a three‐dimensional polymer comprising numerous phenylpropane units interconnected by irregular C‐O bonds (β‐O‐4, α‐O‐4) and C‐C bonds (5‐5, β‐β, β‐1, β‐5). Remarkably, lignin stands as the sole substance containing benzene rings among renewable resources.^[20]^ The fundamental structural units of lignin encompass Parahydroxyphenylpropane, syringylpropane, and guaiacylpropane, which serve as precursors corresponding to coumarin alcohol, coniferyl alcohol, and sinapyl alcohol, respectively. These alcohol compounds intricately assemble into complex three‐dimensional structures via diverse bonding arrangements. Predicated on the composition and linkage structure of these alcohol units, lignin is categorized into softwood lignin, hardwood lignin, and herbaceous lignin, as illustrated in Figure 3.^[21]^ Lignin forms bonds with cellulose and hemicellulose, predominantly via ether bonds, and particularly through ester bonds with hemicellulose, thereby engendering rigid structures that shield polysaccharides from bacterial assault and chemical or enzymatic hydrolysis. However, this very characteristic presents challenges for the effective valorization of lignocellulose.

## 3 LIGNOCELLULOSE PRETREATMENT

In lignocellulosic biomass, the three primary components—cellulose, hemicellulose, and lignin—interlace and chemically bond, yielding a complex three‐dimensional structure. These inherent characteristics confer high biological and chemical stability upon lignocellulosic fibers impeding their conversion and utilization. It is acknowledged that enzymatic hydrolysis without prior pretreatment of lignocellulose may only achieve a maximum theoretical yield of 20% of reducing sugars.^[22]^ Hence, effective treatment methods are imperative to unravel the cross‐linked structure of cellulose, hemicellulose, and lignin, dismantle their biological barriers, facilitate component breakdown and separation, and enable chemical reagents or hydrolytic enzymes to efficiently interact with substrates for biochemical conversion, laying the groundwork for the comprehensive utilization of these major components. The primary challenge lies in devising cost‐effective, environmentally friendly, and sustainable production methods for generating high‐selectivity and high‐yield target chemicals. Presently, the predominant lignocellulose pretreatment technologies are broadly classified into four categories: physical, chemical, physicochemical, and biological methods.

### 3.1 Physical pretreatment

Physical treatment methods typically encompass techniques that apply mechanical energy and internal energy to biomass materials, thereby altering their physical properties and microstructure. Traditional methods, such as grinding, extrusion, cutting, and shredding, are commonly employed to modify biomass particle size, surface area, crystallinity, and molecular polymerization degree. These methods offer advantages such as convenient processing, short cycles, absence of introduced chemical reagents or other exogenous substances, environmental friendliness, and low production costs. For instance, Zakaria et al.^[23]^ utilized palm tree branches as raw materials and enzymatic hydrolysis to produce glucose. Ball milling reduced the crystallinity of the samples from 56.1% to 9.3%, resulting in a glucose yield increase to 67.5% after cellulase hydrolysis, compared to only 15.9% for untreated raw materials. Similarly, Yoo et al.^[24]^ demonstrated that increasing the screw speed during extrusion pretreatment of soybean hulls enhanced glucose yield by reducing biomass fiber length and increasing enzyme contact surface area. However, mechanical treatment often entails higher energy consumption compared to the inherent energy of biomass, resulting in limited sugar release, restricted commercial feasibility, and failure to remove lignin, which impedes enzyme access to cellulose and inhibits cellulase action.

With the continuous advancement of science and technology, microwave, ultrasound, and high‐energy radiation technologies have emerged as alternative biomass pretreatment approaches. Microwave radiation exerts a thermal effect that rapidly disintegrates biomass raw material structures, enhancing cellulose accessibility and reactivity.^[25]^ Ultrasound technology, characterized by efficient, environmentally friendly, and safe pretreatment, induces cavitation in liquids via ultrasound waves.^[26]^ Cavitation disrupts the surface structure of lignocellulosic biomass and weakens chemical bonds, thereby reducing molecular polymerization and transforming insoluble macromolecules into soluble small molecules. High‐energy electron radiation pretreatment exploits physical and chemical changes induced by ionizing radiation from radioactive isotopes and accelerator‐produced ion beams.^[27]^ These pretreatment methods offer advantages such as rapid and uniform heating, short reaction times, low reaction activation energy, minimal by‐products, high efficiency, energy savings, and minimal to no pollution. However, when processing large quantities of products, the low penetration rate and uneven power distribution of radiation may result in reduced sugar content. Therefore, the primary application of radiation energy waves focuses on enhancing the efficiency of other traditional pretreatment methods, such as ultrasound‐assisted alkali, acid, organic solvent, hydrolysis, and ionic liquid treatments. For instance, Mikulski et al.^[28]^ observed that combining microwave radiation with dilute acid and cellulase pretreatment techniques increased the glucose yield from wheat and rye distillation. Pretreatment with 300 W microwave energy for 15 min resulted in a high glucose concentration of 156 mg/g.

### 3.2 Chemical pretreatment

Chemical methodologies for biomass treatment are recognized for their cost‐effectiveness, efficiency, and capacity to selectively control specific properties, rendering them extensively utilized techniques for pretreating lignocellulosic biomass. These methods encompass acid, alkali, ionic liquids, deep eutectic solvents (DESs), organic solvents, and oxidants, each with distinctive targeting and characteristics. Fundamentally, these agents introduce chemical energy to alter the chemical bonds and functional groups of lignocellulosic biomass, thereby regulating its physical structure and chemical composition. For instance, modifying the crystallinity of cellulose, enhancing the specific surface area of biomass, and eliminating lignin and hemicellulose contribute to mitigating the recalcitrance of lignocellulosic biomass and augmenting conversion efficiency. However, chemical treatment methods inevitably introduce toxic reagents into the system, leading to product loss during separation and washing processes, and may also produce toxic and harmful pollutants. Overcoming the challenge of achieving zero emissions of pollutants through continuous processes is a major issue in chemical treatment methods.^[29]^

Acid hydrolysis stands out as one of the premier methods for dissolving hemicellulose and rendering cellulose more accessible. This process involves the hydrolysis of hemicellulose and the condensation/precipitation of lignin, resulting in the transformation of polysaccharides into oligosaccharides and monosaccharides by breaking glycosidic bonds, thus disrupting the structure of lignocellulose.^[30]^ Acid pretreatment techniques excel in disintegrating lignocellulose and converting hemicellulose compared to many conventional methods. The most widely used forms of acids include dilute and concentrated acids. Acid concentration and reaction temperature are two key parameters governing the extent of hemicellulose removal and residual cellulose porosity.^[31]^ Although dilute acid conditions necessitate higher reaction temperatures (>200°C), resulting in increased energy consumption, strong acid conditions yield lower reaction temperatures but generate fermentation inhibitors and entail high equipment requirements. For these reasons, dilute acid pretreatment has emerged as an economically effective alternative for biomass processing.

Recent years have witnessed significant progress in the research on alkaline pretreatment of lignocellulosic feedstock for the production of high‐value chemicals and biomass energy.^[32]^ Alkaline pretreatment operates on the saponification reaction of ester groups on hemicellulose and lignin, leading to the partial dissolution of these components. Additionally, the degree of polymerization and crystallinity of cellulose decrease post alkaline pretreatment, thereby enhancing the accessibility of enzymes to lignocellulosic feedstock. Potassium, sodium, ammonium, and calcium hydroxides are among the most commonly employed alkalis. Haque et al.^[33]^ demonstrated that pretreating barley straw with dilute sodium hydroxide was effective, yielding a glucose output of 86.7% after enzymatic hydrolysis. Treating at 105°C for 10 min with 2% NaOH resulted in an 84.8% lignin removal rate. Similarly, Wu et al.^[34]^ achieved 85% glucose yield during cellulase hydrolysis of sugarcane bagasse following treatment with a 1–5 mol/L sodium hydroxide solution for 16 h. Compared to acid pretreatment methods, alkaline pretreatment obviates the need for specialized corrosion‐resistant reaction equipment and generates lower levels of by‐products post‐pretreatment, albeit with higher material consumption.

In recent years, there has been extensive research on the utilization of ionic liquids for pretreating lignocellulosic biomass, owing to their capacity for hydrogen bond coordination, which enables the complete dissolution of biomass. Ionic liquids, typically composed of large organic cations and small inorganic anions, exhibit favorable chemical structures and thermal stability, maintaining a liquid state at low vapor pressures and across a wide temperature range. Common organic cations encompass imidazole, amino, pyrrole, pyridine, sulfonamide, and phosphate groups. Presently, imidazolium‐based ionic liquids are predominantly employed in lignocellulosic biomass pretreatment.^[35]^ During pretreatment, crystalline cellulose undergoes conversion into an amorphous structure, while the phenolic hydroxyl groups in lignin increase, resulting in a reduction in molecular weight and facilitating the extraction and separation of polysaccharides and lignin.^[36]^ However, ionic liquids are characterized by high viscosity, complicating recovery, and exhibiting toxicity to enzymes, thereby impeding subsequent processing. Achieving large‐scale industrial application of ionic liquids still poses significant development challenges. Over the past two decades, in response to conventional solvent pollution and toxicity concerns, the development of environmentally friendly and non‐toxic green solvents, such as DESs, with physicochemical properties akin to ionic liquids has gained widespread attention. Compared to traditional solvents and ionic liquids, DESs are economical, non‐toxic, biodegradable solvents that are straightforward to synthesize and do not necessitate complex purification processes. Comprising hydrogen bond donors (HBD) and acceptors (HBA), DESs have melting points lower than those of individual compounds.^[37]^ HBDs include glycerol, ethylene glycol, 4‐hydroxybenzaldehyde, and some organic acids, while HBAs encompass acetamide, choline chloride, and betaine.

Research indicates that treating lignocellulosic feedstock with organic solvents such as methanol, ethanol, and acetone effectively eliminates lignin and partially dissolves hemicellulose to some extent, thereby leading to an enhanced enzymatic conversion efficiency of cellulose in the pretreated samples. Furthermore, the addition of acidic catalysts (sulfuric acid, hydrochloric acid, or oxalic acid) during organic solvent pretreatment can effectively augment the dissolution of hemicellulose.^[38]^ In comparison to other chemical pretreatment methods, the primary advantage of organic solvent pretreatment lies in its capability to recover lignin as a byproduct. Nonetheless, the cost of organic solvent pretreatment for biomass feedstock is relatively high, and the process necessitates equipment capable of withstanding high pressure due to the requirement for high‐pressure treatment, further escalating the cost of pretreatment. For the efficient removal of lignin from lignocellulosic biomass, oxidants such as hydrogen peroxide, ozone, or oxygen can also be employed. By disrupting the phenolic ring structure of lignin, these oxidants facilitate acidic pretreatment techniques. Alkaline hydrogen peroxide has been widely utilized in recent years due to its high lignin removal efficiency in biomass. Under normal conditions, hydrogen peroxide solely reacts with the aliphatic portion of lignin, but under alkaline conditions, it reacts with phenolic compounds in lignin. Through the dual action of alkaline hydrogen peroxide pretreatment, the enzymatic digestibility of the samples after pretreatment is significantly enhanced.^[39]^

### 3.3 Physical‐chemical pretreatment

Steam explosion pretreatment involves subjecting lignocellulosic feedstock to high‐temperature and high‐pressure steam.^[40]^ Initially, the lignocellulosic biomass powder is immersed in high‐pressure steam at temperatures ranging from 160 to 270°C for several seconds to minutes, followed by a sudden release of pressure to atmospheric pressure. During the high‐pressure phase, the elevated‐temperature steam infiltrates the biomass, leading to significant degradation of hemicellulose. The high temperature induces water ionization and acetyl group formation, with the resultant acetic acid promoting lignocellulose hydrolysis. Upon pressure release, rapid water evaporation disrupts the lignocellulosic structure, facilitating lignin separation. Steam explosion has proven effective in enhancing lignocellulosic biomass feedstock conversion into fuel ethanol. For instance, Rochón et al.^[41]^ treated eucalyptus sawdust with steam explosion at 200°C for 10 min, resulting in substantial hemicellulose degradation into xylose and a minor fraction of oligoxyloses. The solid residue post‐steam explosion underwent enzymatic hydrolysis and fermentation to yield ethanol at 259 L/t, effectively harnessing eucalyptus components. Analogous to steam explosion, hydrothermal pretreatment utilizes water as a pretreatment agent sans additional chemical reagents. At elevated temperatures, hydronium ions can degrade glycosidic bonds and acetyl groups in hemicellulose, with ensuing acetic acid further catalyzing hemicellulose hydrolysis to yield oligosaccharides and monosaccharides. Following hydrothermal pretreatment, two products are obtained: a solid residue enriched in cellulose and lignin, and a liquid rich in degraded sugars from hemicellulose.^[42]^ However, in comparison to steam explosion, hydrothermal pretreatment necessitates higher water and energy consumption, yielding lower product concentrations. In summary, steam explosion and hydrothermal pretreatment operate on similar principles and are both deemed environmentally friendly pretreatment methodologies.

The ammonia fiber explosion (AFEX) pretreatment method involves subjecting biomass to liquid ammonia (1:1 w/w) in a reactor under moderate reaction temperatures (60–170°C) and high pressure (15–30 bar) for a brief duration (5–60 min). Subsequently, the heating device is deactivated, and the ammonia within the reaction is released to yield dried material. Although residual ammonia may persist in the material, it can serve as a nitrogen source for the ethanol fermentation of monosaccharides in the hydrolysis liquid. Throughout the AFEX pretreatment process, ammonia interacts with the ester bonds between lignin and hemicellulose as well as the chemical bonds in lignin, yielding corresponding acetamide and aminophenol compounds. Liquid ammonia has the capability to dissolve some lignin, and post‐pretreatment both liquid ammonia and ammonia gas are liberated, thereby leaving behind numerous nanoscale pores in the cell walls, which facilitate the contact of cellulase enzymes.^[43]^ Additionally, ammonia's high volatility permits the recovery of ammonia gas post‐pretreatment, which can then be recycled for cost reduction and environmental conservation purposes. Crop residues produce fewer inhibitors during the AFEX pretreatment process, enabling the pretreated material to undergo hydrolysis and fermentation without detoxification. However, the efficacy of AFEX pretreatment on biomass with high lignin content falls short of expectations.

### 3.4 Biological pretreatment

Biological pretreatment stands out as the most natural, environmentally friendly, and cost‐effective approach to biomass processing. It typically requires no energy or chemical reagents, thus circumventing the need for expensive equipment. This method entails the introduction of fungi (e.g., white rot fungi, brown rot fungi, soft rot fungi) or enzymes (such as ligninase and peroxidase) to degrade lignin or hemicellulose in lignocellulosic biomass. This degradation disrupts the dense structure formed by cellulose, hemicellulose, and lignin, thereby facilitating the conversion and utilization of these components in subsequent processes.[^44^, ^45^] Fungi degrade biomass through specific enzymes produced during metabolic activities. Compared to microorganism introduction, enzymatic pretreatment obviates the need for microbial metabolism processes and raw material consumption. Biological pretreatment offers environmental friendliness, low energy consumption, and absence of fermentation inhibitors (e.g., furfural and 5‐hydroxymethylfurfural [HMF]). Nevertheless, its long processing time and low production efficiency have steered its development trend towards combination with other pretreatment methods.

## 4 BIOMASS CONVERSION

There are three primary methods for converting lignocellulose: direct combustion, thermochemical conversion, and biochemical conversion.^[46]^ Direct combustion entails burning lignocellulose to generate electricity. However, due to biomass's high oxygen content and low calorific value, combustion efficiency is suboptimal, resulting in underutilization of biomass resources. Thermochemical conversion employs gasification, pyrolysis, and hydrolysis to dismantle the dense structure of lignocellulose, decreasing polymerization and oxygen content to yield small molecule products and enhancing the biomass value. Biomass gasification entails breaking down biomass polymers and reconstituting them under specific temperature conditions with air, oxygen, or steam to selectively convert them into high‐quality combustible gases like carbon monoxide, hydrogen, and light hydrocarbons. Pyrolysis typically involves the high‐temperature conversion and decomposition of biomass feedstocks in an anaerobic or low‐oxygen environment, yielding char, bio‐oil, and gaseous products. Hydrolysis entails breaking down lignocellulose into smaller molecules with water assistance, catalyzing the conversion process. Biochemical conversion employs microorganisms and enzymes to catalyze lignocellulose conversion, yielding small molecule chemicals such as methane and ethanol via selective cleavage and fermentation processes. Biomass conversion products exist in gas, liquid, and solid phases. Gases can serve as fuel or be utilized to produce H_2_ through catalytic reforming for applications like methanol synthesis. Liquids, mainly comprising bio‐oil and hydrolysis products, contain over a hundred organic compounds and can be directly used as fuel and in the food industry or serve as chemical intermediates for further processing into higher‐value chemicals and biofuels.^[47]^ Chemicals, from an added value perspective, offer superior cost‐effectiveness compared to liquid fuels. Solid char, predominantly carbon‐based, exhibits high stability and features a complex pore structure. Additionally, it contains abundant oxygen‐containing groups on its surface, rendering it useful in various applications such as soil improvement and remediation, adsorbents, and the preparation of carbon‐based materials for industrial and material chemistry purposes.

In biomass processing and conversion, catalytic reactions play a pivotal role, continually enhancing biomass value through diverse catalytic pathways. Two prevalent catalytic methodologies are homogeneous catalysis and heterogeneous catalysis. Homogeneous catalysts encompass mineral acids, organic acids, transition metal chlorides, ionic liquids, and high‐pressure CO_2_ among others.^[48]^ Homogeneous catalytic systems involve catalysts and reactants in the same phase, facilitating precise control over reaction conditions and achieving high selectivity. However, this coexistence also complicates catalyst recovery and reusability. Heterogeneous catalysts comprise zeolites, resins, metal oxides, metal phosphates, carbon‐based solid acids, heteropolyacids, clays, and coordination polymers among others.[^49^, ^50^] In heterogeneous catalysis, catalysts and reactants exist in distinct phases, simplifying separation and post‐reaction processing. Nonetheless, heterogeneous catalysts often exhibit slower reaction rates, encounter greater mass transfer limitations between reactants and catalysts, and entail relatively intricate synthesis and recovery procedures, resulting in elevated costs. The catalytic mechanisms predominantly involve Bronsted acid sites, Lewis acid sites, and metal active sites, which achieve efficient product selectivity through the distinct strengths and synergistic effects of these active sites. Bronsted acid sites facilitate dehydration and decarboxylation reactions, whereas Lewis acid sites tend to catalyze isomerization, aldol condensation, hydride shifts, and ring‐opening reactions. The acidity and basicity strengths significantly impact the performance of diverse catalysts. Hydrogenation, hydrolysis, HDO, and oxidation reactions are often modulated by metal active sites.

### 4.1 Cellulose/hemicellulose conversion

Cellulose and hemicellulose, following diverse pretreatments and conversions, yield a spectrum of intermediate derivative products, comprising various oxygen‐containing C2–C6 platform chemicals, commonly termed as biomass platform molecules. Biomass platform molecules are characterized as substrate chemicals capable of generating a plethora of high‐value products and can be further refined into premium chemicals and polymer materials. In 2004, the U.S. Department of Energy delineated 12 chemical constituents attainable from biomass as potential platform chemicals.^[51]^ Subsequently, in 2010, Bozell and Petersen of the U.S. Department of Energy revised the inventory of platform chemicals, judiciously incorporating certain furan compounds, biomass‐based sugars, and carboxylic acid compounds.^[52]^ Specifically, these encompass furan compounds and their derivatives (furfural, HMF, 2,5‐furandicarboxylic acid), organic acid compounds (levulinic acid, 3‐hydroxypropionic acid, succinic acid [SA], lactic acid [LA]), polyol compounds (glycerol, ethanol, sorbitol, xylitol), and other constituents (biomass alkanes).

#### 4.1.1 Furfural

**FIGURE 4:**
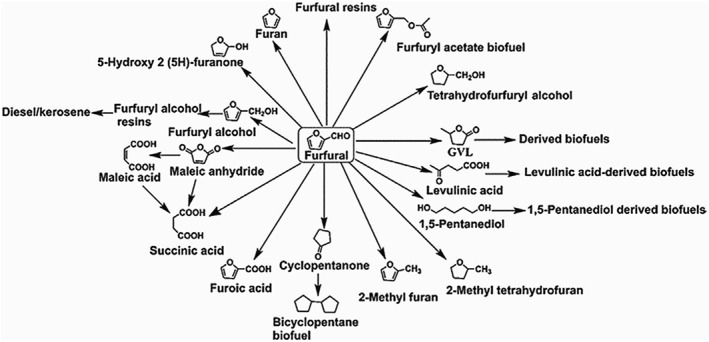
Value‐added chemicals synthesized from furfural. Reproduced with permission.^[55]^ Copyright 2019, Elsevier.

Furfural is a dehydration product of xylose and xylan formed from a furan ring substituted with a formyl group. Currently, the production of furfural faces issues such as low yield, the reliance on homogeneous acid catalysts, high thermal energy consumption, and severe pollution from acidic wastewater. Namhaed et al.^[53]^ utilized formic acid as a catalyst instead of traditional inorganic acids, mitigating equipment damage while employing environmentally friendly supercritical carbon dioxide for furfural extraction, thereby achieving higher yields and preventing furfural degradation. Under conditions of 140°C and 20 MPa, with a constant flow rate of CO_2_ at 5 g/min, an initial concentration of 10 g/L xylose, and 10 wt% formic acid, the highest furfural yield reached 68.5% after 5 h of reaction (selectivity was 71.4%, and separation efficiency was 99%). To optimize experimental methodologies, solid acid catalysts (such as zeolites, ion exchange resins, transition metal oxides, and mesoporous silicates) along with DESs have been extensively investigated. Bruce et al.^[54]^ were the first to use a small‐pore zeolite catalyst (SAPO‐34) in a γ‐valerolactone (GVL)/water single‐phase system, achieving a furfural yield of 40% from xylose, thus demonstrating the feasibility of small‐pore zeolites. Additionally, furfural has significant applications: it can be utilized in the production of fuels, resins, and lubricants, and as a precursor for high‐value chemicals (as shown in Figure 4).^[55]^ The aldehyde and furan ring in furfural enable the production of nearly 100 chemicals through various reactions (such as acetalization, acylation, aldolization, reduction, oxidation, amination, alkylation, halogenation, and nitration), including maleic acid, SA, maleic anhydride, furfuryl alcohol, furfurylamine, cyclopentanone, and GVL.^[56]^ Among these, furfuryl alcohol is the most significant chemical derived from furfural, accounting for 65% of downstream furfural products. Furfuryl alcohol can be converted into various chemicals such as ethyl furfuryl ether, acetylpropionic acid, tetrahydrofurfuryl alcohol, and GVL. Furfuryl alcohol is produced through the catalytic hydrogenation of furfural, compared to gas‐phase hydrogenation, liquid‐phase hydrogenation can achieve higher yields of furfuryl alcohol. Zhang et al.^[57]^ utilized methanol as an effective hydrogen donor and calcium oxide‐supported Raney Ni as a catalyst, achieving furfural conversion and furfuryl alcohol yields of 100% and 99%, respectively.

#### 4.1.2 5‐Hydroxymethylfurfural

**FIGURE 5:**
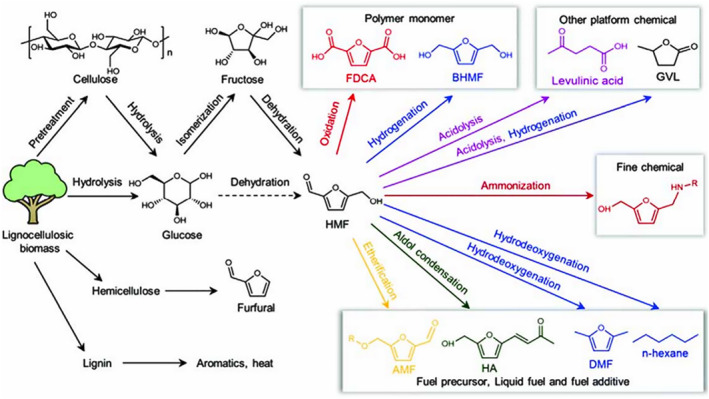
Value‐added chemicals synthesized from 5‐HMF. Reproduced with permission.^[61]^ Copyright 2021, Royal Society of Chemistry. 5‐HMF, 5‐hydroxymethylfurfural.

HMF is an important furan‐based compound that can be converted into a range of high‐value chemicals, biofuels, and polymer monomers. Compared to furfural, the raw materials for producing HMF are more abundant and derived from various monosaccharides (fructose, glucose), disaccharides (sucrose, maltose), and polysaccharides (inulin, starch, cellulose).^[58]^ The primary reactions involve fructose dehydration or glucose isomerization to fructose, with disaccharides and polysaccharides first hydrolyzing to fructose or glucose. Typically, water or organic solvents present limitations in HMF production due to by‐product formation. Consequently, researchers have explored various solvent systems, including ionic liquids and DESs. Prasad et al.^[59]^ used [Bmim]Cl as the reaction solvent and ZSM‐5 zeolite as the catalyst, achieving a 95% fructose conversion and a 67.2% HMF yield after 90 min at 110°C. However, ionic liquids present issues with recyclability, high cost, and environmental pollution, making DESs more advantageous. Karimi et al.^[60]^ employed a DES composed of oxalic acid and malonic acid as an acid catalyst, achieving fructose dehydration with a 97% HMF yield in just 1 min at 90°C, the fastest reported process to date. Due to its active aldehyde, hydroxymethyl, and furan ring functional groups, HMF can be converted into various high‐quality liquid fuels and chemical products through etherification, oxidation, and hydrogenation, as illustrated in Figure 5.^[61]^ For instance, HMF can be oxidized to 2,5‐furandicarboxaldehyde and 2,5‐furandicarboxylic acid, which are utilized to synthesize pharmaceutical intermediates, plasticizers, and antibacterial agents, as discussed in the next section. Through hydrogenation, HMF can be converted into 2,5‐dihydroxymethylfuran, 2,5‐dimethylfuran, 2,5‐dihydroxymethyltetrahydrofuran, and 2,5‐dimethyltetrahydrofuran, which serve as gasoline additives and biomass‐based fuels. Among these, 2,5‐dihydroxymethylfuran is especially noteworthy. The main challenge in achieving high‐selectivity hydrogenation of 2,5‐dihydroxymethylfuran lies in suppressing side reactions such as furan ring hydrogenation and ring opening as well as hydroxyl hydrogenolysis. He et al.^[62]^ investigated Pt nanoparticles supported on manganese oxide octahedral molecular sieves (Pt/OMS‐2), which exhibited excellent selectivity in the aqueous‐phase hydrogenation of HMF to 2,5‐dihydroxymethylfuran, avoiding ring hydrogenation or opening even under harsh conditions. At 30°C and 1.5 MPa H_2_, a 97.6% yield of 2,5‐dihydroxymethylfuran was achieved within 2 h.

#### 4.1.3 2,5‐Furandicarboxylic acid

**FIGURE 6:**
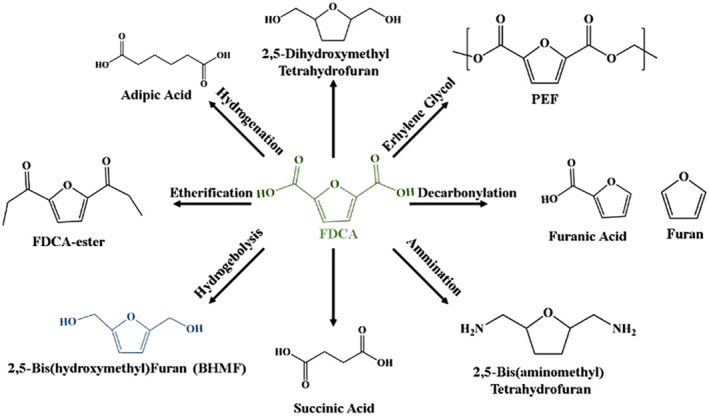
Value‐added chemicals synthesized from FDCA. Reproduced with permission.^[63]^ Copyright 2013, ACS Publications. FDCA, 2,5‐furandicarboxylic acid.

2,5‐Furandicarboxylic acid (FDCA) is the only bio‐based platform compound with an aromatic ring that is planar and has a rigid structure. It can be synthesized into important chemical intermediates, material intermediates, and pharmaceutical intermediates such as 2,5‐bis(aminomethyl)tetrahydrofuran and adipic acid through reactions like hydrogenation, acylation, and ring‐opening, as illustrated in Figure 6.^[63]^ Based on the different starting materials, the synthesis routes for FDCA can be divided into five pathways: furanoic acid disproportionation, hexose diacid, furanylcarbonylation, diglycol, and HMF oxidation. Among these, HMF, derived from low‐cost and widely available bio‐based carbohydrates, offers the highest cost advantage and higher yield of the target product with fewer side effects compared to other raw materials. The synthesis of FDCA from HMF can be further categorized into methods such as oxidation with strong oxidants, enzymatic oxidation, electrochemical oxidation, and thermochemical oxidation. Among these, the thermochemical oxidation method is currently the most common for preparing FDCA.^[64]^ For example, Liu et al.^[65]^ prepared Pt NPs catalysts for the oxidation of HMF to FDCA, achieving a 95.1% yield of FDCA under optimized conditions (100°C, 10 h, 75 mL/min O_2_). Additionally, FDCA is primarily used to replace the benzene ring series in the eight major petrochemical platform compounds (benzene, toluene, xylene, ethylene, propylene, butadiene, methanol, and naphthalene), facilitating the production of new bio‐based polymer materials with superior performance through polymerization with monomers such as diols and diamines.^[66]^ It is considered as a direct substitute for terephthalic acid and adipic acid in polyester and other polymer production, reducing fossil fuel consumption. Compared to traditional polyethylene terephthalate materials, polyethylene furanoate, synthesized from FDCA and ethylene glycol, exhibits superior thermodynamic properties and barrier characteristics, and also offers the advantages of being non‐toxic, renewable, and biodegradable. This gives it significant potential for application in the plastics and resin industries.

#### 4.1.4 Levulinic acid

**FIGURE 7:**
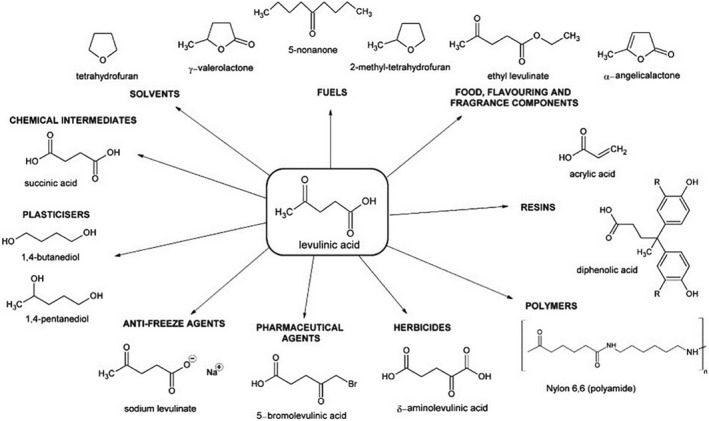
Value‐added chemicals synthesized from levulinic acid. Reproduced with permission.^[67]^ Copyright 2011, Wiley Online Library.

Levulinic acid is a linear C5 alkyl carbon chain containing a ketone and a carboxyl group, and it is a promising platform chemical molecule widely used in fuel additives, herbicides, pharmaceuticals, flavorings, surfactants, and more. Currently, levulinic acid is mainly produced through the following two routes. The first route uses C6 sugars (glucose, fructose, mannose, and galactose) as raw materials, which undergo acid‐catalyzed isomerization, dehydration, and hydration reactions. The second route involves the acid‐catalyzed hydrolysis of furfural to produce levulinic acid. Since the second method requires C5 sugars (xylose and arabinose) to be hydrolyzed to produce furfural, which is then further hydrogenated, the first method is more economically efficient and suitable for large‐scale industrial production. Additionally, levulinic acid can be converted into biofuel derivatives such as 2‐methyltetrahydrofuran, GVL, and alkyl levulinates, as shown in Figure 7.^[67]^ Among these, GVL is the most extensively studied. In the hydrogenation process, common metal catalysts include Ru, Pd, Pt, Ni, Rh, Co, and Au. For example, Barla et al.^[68]^ prepared Co‐loaded zeolite catalysts for the hydrogenation of levulinic acid, achieving a levulinic acid conversion rate of 99% and a GVL selectivity of over 80% at low reaction temperatures. To streamline the process, formic acid, a byproduct of levulinic acid production, can be used as a hydrogen source to directly convert cellulose to GVL. Zhu et al.^[69]^ efficiently converted cellulose into the value‐added product GVL in one step. They prepared CoNi nanoparticles supported on N‐doped ultrathin graphene sheets combined with Ag^+^, achieving a GVL yield of 55.9%. Moreover, compared to H_2_, formic acid can reduce levulinic acid faster due to its low dissociation barrier and high dissociation rate.

#### 4.1.5 3‐Hydroxypropionic acid

3‐Hydroxypropionic acid (3‐HP) is a weak organic acid with high solubility, easily dissolving in water, alcohol, ether, and other solvents. Due to the presence of both hydroxyl and carboxyl groups, it possesses reactive chemical properties, making it easily convertible into various important chemicals or applicable in the development of biodegradable new materials.^[70]^ There are mainly two methods for synthesizing 3‐HP: chemical synthesis and microbial synthesis. The chemical synthesis method involves expensive and toxic raw materials, harsh reaction conditions, significant pollution, and high equipment costs, limiting its future application prospects. On the other hand, microbial synthesis uses cheap carbon sources (glucose and glycerol) as raw materials catalyzed by various bacteria and enzymes, resulting in minimal environmental impact and good development prospects. To achieve high yield, high titer, and high productivity, metabolic engineering and genetic engineering are widely used in research and production due to the inability of natural microorganisms to meet these conditions. Chen et al.^[71]^ expressed the gpd and gpp genes from *Saccharomyces cerevisiae* in *Corynebacterium glutamicum*, first converting glucose to glycerol, and then using aldehyde dehydrogenase genes from different sources to synthesize 3‐HP, achieving a yield of 38.6 g/L. Subsequently, to expand the substrate range, they introduced xylose metabolism genes into the host bacteria, allowing simultaneous fermentation of glucose and xylose, thereby improving the yield of 3‐HP. 3‐HP has three main applications: First, due to its antibacterial activity, it can be used directly as an additive or preservative in food or feed production. Second, 3‐HP can generate lactones, polyesters, and oligomers, such as poly(3‐hydroxypropionate), which exhibit good tensile and elongation properties and biodegradability, making it an excellent material to replace plastics. Additionally, 3‐HP can be used as an intermediate to synthesize resins, fibers, adhesives, and more. Third, 3‐HP serves as a substrate for synthesizing various industrially important C3 chemicals through various redox reactions, producing 1,3‐propanediol, acrylic acid (AA), malonic acid, acrylamide, acrylonitrile, etc., widely used in adhesives, plastic packaging, fibers, detergents, and more. Among these, AA has significant market potential.^[72]^ Li et al.^[73]^ studied the dehydration of 3‐HP to AA using different acidic catalysts, finding that silica gel, which contains only a small amount of L acid sites, achieved the highest AA yield of over 99.0%. In contrast, catalysts containing B acid sites led to the formation of by‐product acetic acid.

#### 4.1.6 Succinic acid

SA, also known as butanedioic acid, is a common natural organic acid found in amber as well as in humans, animals, plants, and microorganisms, playing a significant role in biological metabolism. Currently, chemical methods for producing SA include paraffin oxidation, catalytic hydrogenation, and electrolytic reduction. However, these methods suffer from high costs, energy consumption, and severe pollution, thus making the biological production of SA widely attractive. For instance, Klasson et al.^[74]^ used a genetically engineered *Escherichia coli* strain AFP184 to convert all sugars (glucose and fructose) in hydrolysates from sorghum into SA and acetic acid. The final concentration of SA was 27 g/L, but the results indicated that using pure glucose could produce higher levels of SA (60 g/L), though it still does not meet industrial production requirements. SA serves as a platform molecule for producing various important chemicals, including γ‐butyrolactone, 1,4‐butanediol, tetrahydrofuran, and maleic anhydride with broad applications in bioplastics, cosmetics, pharmaceuticals, and the food industry.^[75]^ SA esters are green and environmentally friendly plasticizers that can replace toxic phthalate esters. They are not only less toxic but also demonstrate better resistance to migration within polymer matrices. Elsiwi et al.^[76]^ synthesized di‐n‐heptyl succinate, a green degradable plasticizer for Polyvinyl chloride, through an esterification reaction catalyzed by sulfuric acid using SA and n‐heptanol. Furthermore, optimizing the production process of SA derivatives can significantly reduce production costs. Chen et al.^[77]^ improved a niobium acid catalyst through acid treatment, achieving a succinic anhydride yield of 80% from SA dehydration at 150°C, greatly promoting industrial development.

#### 4.1.7 Lactic acid

**FIGURE 8:**
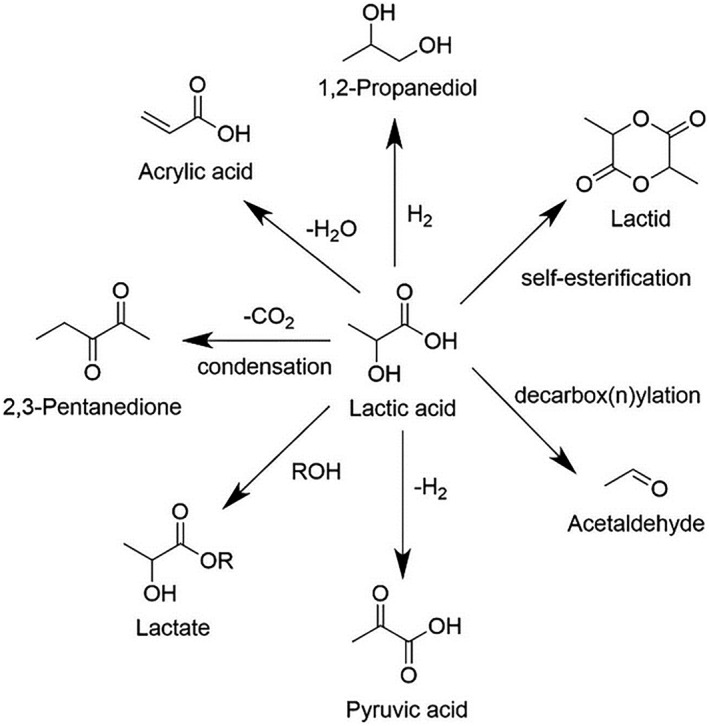
Value‐added chemicals synthesized from lactic acid.

LA, also known as 2‐hydroxypropanoic acid, is an important organic acid used in the production of dairy products, food additives, and chemicals. It finds applications in the pharmaceutical and cosmetic industries as well. The key to producing LA from cellulose‐derived sugars involves catalyzing reverse aldol condensation and isomerization, with Lewis acid sites playing a crucial role. Zhang et al.^[78]^ synthesized an Sn‐Beta zeolite catalyst via a hydrothermal method, which catalyzes the conversion of xylose to LA with a yield of 70% at 200°C for 1 h. In this catalytic process, Sn in the Si‐O‐Sn framework of the zeolite acts as a Lewis acid site, facilitating both coupling and cleavage of C‐C bonds. Direct catalytic production of LA from cellulose has garnered significant attention due to the challenges of hydrolysis and the propensity for self‐polymerization. Verma et al.^[79]^ developed a Ga‐doped Zn/HNZY zeolite catalyst capable of converting cellulose directly to methyl lactate in one step under supercritical conditions, achieving approximately 54% methyl lactate yield at 280°C for 3 h. Glycerol, a byproduct of biodiesel, presents promising applications and economic value for conversion into LA. Ftouni et al.^[80]^ synthesized a Pt/ZrO_2_‐supported catalyst that catalyzes glycerol to LA in an alkaline medium under 3 MPa helium atmosphere, yielding 80% LA at 180°C for 8 h. Recently, polylactic acid (PLA) synthesized from LA monomers has gained significant attention due to its complete biodegradability and thermoplastic nature, offering a sustainable alternative to non‐biodegradable plastics in various packaging applications such as courier bags, food packaging, and shopping bags.^[81]^ PLA synthesis methods include direct polymerization and ring‐opening polymerization of lactide. Each method has its advantages and disadvantages: direct polymerization is straightforward but faces challenges in water removal, limiting molecular weight, while ring‐opening polymerization of lactide can produce high molecular weight PLA but is complex and costly industrially. Additionally, PLA exhibits excellent biocompatibility as LA is a normal metabolic product in the human body, making PLA suitable for biomedical applications such as catheters, drug carriers, tissue regeneration scaffolds, and absorbable implants required in orthopedic surgery.^[82]^ LA, containing a hydroxyl and a carboxyl group, can be further valorized into high‐value chemicals through various reactions.^[83]^ As shown in Figure 8, LA can be hydrogenated to produce 1,2‐propanediol, oxidized to pyruvic acid, dehydrated to AA, or undergo decarbonylation/decarboxylation reactions to yield acetaldehyde.

#### 4.1.8 Glycerol

**FIGURE 9:**
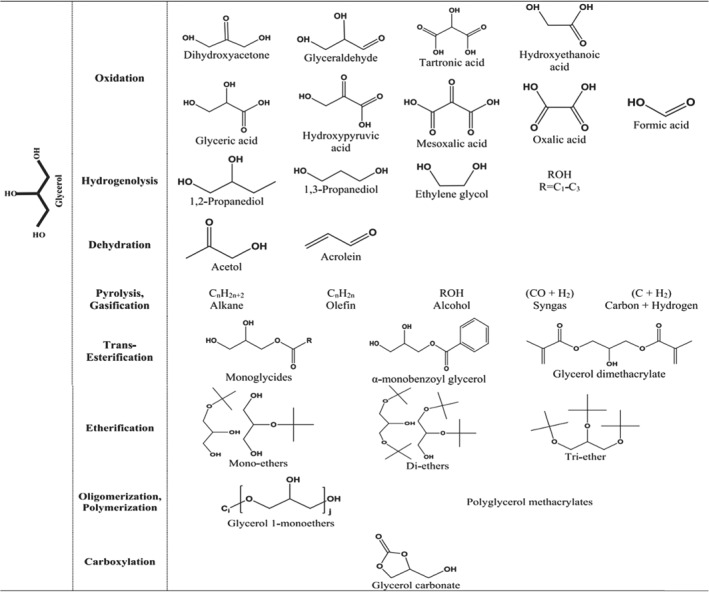
Value‐added chemicals synthesized from glycerol. Reproduced with permission.^[84]^ Copyright 2018, Elsevier.

Glycerol is a typical polyol, non‐toxic, biodegradable, edible, and readily available. It contains two hydroxyl groups with similar properties in its molecule, offering potential for the production of high‐value fine chemicals. Glycerol is a by‐product of biodiesel production, with 0.1 tons generated per ton of biodiesel, leading to a significant surplus of glycerol production capacity. Various reactions such as oxidation, hydrogenation/hydrolysis, esterification, dehydration, pyrolysis, carboxylation, polymerization, and reforming can break and rearrange C‐C, C‐O, and O‐H bonds in glycerol molecules, yielding a plethora of value‐added chemicals, as delineated in Figure 9.^[84]^ For instance, hydrogenation/hydrolysis of glycerol produces 1,3‐propanediol and 1,2‐propanediol, which are applicable in food, pharmaceutical, and industrial sectors. However, selective hydrogenation of the secondary hydroxyl group in glycerol to suppress the formation of 1,2‐propanediol remains challenging. Zuo et al.^[85]^ synthesized a composite oxide Ti‐Si carrier rich in oxygen vacancies, achieving glycerol conversion and 1,3‐propanediol selectivity of 40% and 46%, respectively, after loading with the Pt‐WO_x_ catalyst. Esterification can produce glycerol carbonate, polycarbonates, and polyurethanes with wide applications in textiles, machinery, construction, and defense, albeit currently yielding low production rates. Reforming reactions can yield hydrogen and synthesis gas with hydrogen regarded as a promising clean energy source, while synthesis gas serves as a chemical intermediate to produce various hydrocarbons or liquid hydrocarbon compounds via Fischer‐Tropsch synthesis. The Ni/Al_2_O_3_ catalyst synthesized by Sánchez et al.^[86]^ for glycerol steam reforming achieved over 98% glycerol conversion and 99.7% H_2_ selectivity at 650°C, albeit with limited operational lifespan. Selective oxidation of secondary hydroxyl groups yields dihydroxyacetone, oxidation of primary hydroxyl groups yields glycerol aldehyde, and further oxidation yields glyceric acid, widely used in cosmetics, food, and pharmaceuticals.^[87]^ Wang et al.^[88]^ designed potassium‐doped alkali polymeric carbon nitride to achieve glycerol photocatalytic oxidation with selective co‐production of dihydroxyacetone and glyceric acid. At room temperature, glycerol conversion exceeds 70%, with total selectivity for dihydroxyacetone and glyceric acid also exceeding 70%, demonstrating the feasibility of glycerol photocatalysis under mild conditions. Additionally, these products can further undergo C‐C bond cleavage to produce C1 or C2 products such as ethanol, oxalic acid, formic acid, and CO_2_. In summary, the lack of sufficient yield and selectivity along with stringent reaction conditions and long reaction times hinders the industrial valorization of glycerol.

#### 4.1.9 Ethanol

Bioethanol, with its advantages of renewability, sustainability, and environmental friendliness, reduces dependence on petroleum resources and has become a globally recognized resource for eco‐friendly fuel and a major alternative to fossil fuels. Traditional bioethanol production primarily uses sugars and starches via fermentation, often competing with food resources. Therefore, cellulose‐derived ethanol has garnered considerable attention though it remains in the research phase. Song et al.^[89]^ combined tungstic acid and zirconia‐supported Pt catalysts to directly convert cellulose to ethanol in one step, achieving a yield of only 32%. Ethanol serves as a gasoline additive for energy production through direct combustion and finds extensive applications in medical, food, and agricultural industries. Additionally, ethanol's molecular structure, rich in chemical bonds such as O‐H, C‐H, C‐C, and C‐O, allows for conversion into high‐value chemicals like low‐carbon olefins, aromatics, and gasoline, including ethylene, 1,3‐butadiene, acetaldehyde, ethyl acetate, butanol, hydrogen, higher hydrocarbons, and amine compounds.[^90^, ^91^] Ethylene, crucial in the petrochemical industry, is essential for manufacturing plastics (polyethylene), rubber, fibers, styrene, and epoxyethane. Industrial ethanol‐to‐ethylene processes typically use alumina‐based catalysts known for their high stability, conversion rates (99%), and selectivity (97%). However, the presence of 5%–12% water in bioethanol inhibits reactions, reducing conversion rates, necessitating higher reaction temperatures to overcome this effect and increase energy consumption.^[92]^ Researchers explore alternative catalysts to mitigate energy demands; for instance, Cheng et al.^[93]^ synthesized mesoporous SBA‐15 catalysts using palm oil clinker‐derived sodium silicate, achieving an ethanol conversion rate of 73.33% at an ethanol concentration of 50 wt%, with an ethylene yield of 84.7%, albeit at still elevated temperatures. 1,3‐butadiene serves as a critical monomer in polymer synthesis for rubber and synthetic resin industries and as a raw material for adiponitrile, hexamethylenediamine, and nylon‐66. Current research on the mechanism of ethanol‐to‐butadiene conversion generally posits an aldol condensation mechanism, where ethanol dehydrogenation is a key step. Baylon et al.^[94]^ controlled the basicity and Lewis acid sites of ZnZrO_x_ catalysts by varying the Zn/Zr ratio and sodium doping, effectively suppressing ethanol dehydration, promoting ethanol dehydrogenation and aldol condensation, achieving a 47% selectivity to butadiene.

#### 4.1.10 Sorbitol

In similar polyols, sorbitol is the most economical and widely used sugar alcohol extensively employed in food, cosmetics, toothpaste, and related industries as a sweetener, thickener, moisturizer, structuring agent, and dispersant. It is also a major raw material for producing Vitamin C.^[95]^ Currently, industrial hydrogenation of glucose to sorbitol is well‐established, primarily using Ni‐based catalysts. Sorbitol, a highly functionalized C6 molecule with six hydroxyl groups, undergoes various reactions such as hydrogenation, dehydration, and reforming to produce high‐value downstream products or fuels, transforming into lower carbon C1‐C6 alkanes, polyols, LA, and isosorbide.^[96]^ Among these, polyols include ethylene glycol, 1,2‐propanediol, and glycerol, widely utilized in various applications. Ethylene glycol serves as a crucial petrochemical feedstock for producing polyester fibers, antifreeze, unsaturated polyester resins, lubricants, plasticizers, and non‐ionic surfactants. 1,2‐propanediol is essential for unsaturated polyesters, epoxy resins, polyurethane resins, plasticizers, and surfactants extensively used in coatings and reinforced plastics. For instance, Jia et al.^[97]^ catalyzed selective hydrogenation of sorbitol using Pd‐Cu bimetallic catalysts supported on ZrO_2_ with La(OH)_3_ as a base additive, achieving 100% sorbitol conversion and a total selectivity of 61.7% towards ethylene glycol, 1,2‐propanediol, and glycerol. Recently, transfer hydrogenolysis of sorbitol has emerged as a promising conversion process, utilizing a small fraction of sorbitol as a hydrogen source, albeit demanding precise catalyst control. Zhang et al.^[98]^ employed Ni (hydrogenation)‐Re (hydrogenolysis)/C bimetallic catalysts for sorbitol transfer hydrogenolysis, achieving 91.7% sorbitol conversion at 250°C and 1 MPa N_2_ pressure, with a total selectivity of 46.8% towards ethylene glycol and 1,2‐propanediol. Isosorbide, obtained by removing two molecules of water from sorbitol, possesses unique chemical structure and properties, finding versatile applications. Due to its distinctive chirality, isosorbide serves as an intermediate in synthesizing liquid crystal materials and enhances various high‐performance polymer materials such as polyether, polyester, polyurethane, polyamide, polyimide, and polycarbonate, imparting excellent optical properties, thermal stability, impact resistance, and scratch resistance. For example, Sawada et al.^[99]^ used isosorbide as a primary material to synthesize a novel bio‐based dianhydride and condensed it with other commercial diamines to obtain a series of polyimide resins. Experimental results showed that these polyimide resins exhibit excellent permeability with low refractive index, promising applications in optoelectronics.

#### 4.1.11 Xylitol

Xylitol is a natural pentose sugar alcohol widely present in nature and serves as an intermediate in human metabolism. It finds extensive applications in food, pharmaceuticals, chemicals, and everyday products. There are three main methods for producing xylitol: solid‐liquid extraction, chemical synthesis, and biotransformation. Currently, chemical synthesis is used for industrial production of xylitol, but it faces challenges such as nickel catalyst element loss and complex process requirements, necessitating ongoing optimization efforts. Biotransformation offers a promising alternative with mild reaction conditions and lower production costs compared to traditional chemical hydrogenation methods, attracting significant attention both domestically and internationally. Zhang et al.^[100]^ genetically engineered a strain of Kluyveromyces marxianus by combining the ScGAL2N376F gene from brewing yeast with the NcXYL1 gene from *Neurospora crassa*. They further deleted genes GPD1, KU70, PGI1, and XYL2 to create a yeast strain capable (YZB194) of producing xylitol from both glucose and xylose. Under aerobic conditions at 37°C, strain YZB194 achieved a xylitol concentration of 139.96 g/L from 70 g/L glucose and 140 g/L xylose, with a productivity of 0.83 g/L/h. Subsequent fed‐batch fermentation at 4°C increased xylitol production to 203.57 g/L, with a productivity of 2.99 g/L/h and a xylose yield of 0.99 g/g, demonstrating efficient co‐production from glucose and xylose. Xylitol, with sweetness equivalent to sucrose but only two‐thirds of the caloric content, does not require insulin for metabolism, making it a popular sweetener and sugar substitute for diabetic patients in food products.^[101]^ Moreover, xylitol exhibits antimicrobial properties by inhibiting the growth and acid production of various microorganisms, including *Streptococcus pneumoniae*, *Streptococcus mutans*, *Burkholderia cepacia*, and *Staphylococcus aureus*. Consequently, it is used to prevent dental caries, reduce plaque formation, and treat diseases such as acute otitis media, respiratory infections, allergic dermatitis, and wound infections.^[102]^ As a polyol, xylitol undergoes various reactions. For instance, Liu et al.^[103]^ synthesized NiCu‐SiO_2_ catalysts via the precipitation‐gel method using Ca(OH)_2_ as an alkaline additive for hydrogenating xylitol. The optimal Ni ratio of 1:8 achieved the highest combined yield of ethylene glycol and 1,2‐propanediol up to 81%. The enhanced dispersion of Cu due to Ni doping improved catalyst stability, and NiCu alloy exhibited higher activity compared to individual Ni and Cu metals. Xylitol can also be nitrated to produce efficient explosives (nitroxyl xylitol) and esterified with low‐carbon fatty acids for use as plasticizers.

#### 4.1.12 Biomass alkanes

Bio‐hydrocarbons derived from bio‐oils typically have long carbon chains, high viscosity, low combustion efficiency and cannot be directly used as fuels. They require upgrading through processes like catalytic cracking, catalytic hydrogenation, and isomerization to enhance their quality and modify their properties.^[104]^ Additionally, cellulose derivatives, as mentioned earlier, can be processed via hydrolysis, hydrogenation, and alkylation reactions to produce fuels and chemicals such as gasoline, diesel, aviation fuels, and lubricants. Xi et al.^[105]^ developed a high‐acidity Pt/NbOPO_4_ catalyst for the HDO of the xylitol aqueous phase, yielding C5‐C6 linear alkanes with a 60% yield under fixed‐bed reactor conditions at 250°C and 4.0 MPa H_2_. Shorter‐chain alkanes (C4∼C6) can serve as platform molecules, oxidizing into corresponding alcohols, aldehydes, and acids, further transforming into higher‐value chemicals. Catalytic hydrogenation reduces the oxygen content in bio‐oils and increases the effective hydrogen‐to‐carbon ratio, thereby improving their quality, calorific value, and stability for direct use as fuels or additives in petroleum fuels. Isomerization increases the number of alkane branches, enhances the low‐temperature fluidity of lubricants, octane ratings of gasoline, and reduces the pour point of diesel.

### 4.2 Lignin conversion

Lignin, a byproduct of the paper industry and ethanol production from biomass, is often incinerated directly as fuel, resulting in considerable resource wastage.^[106]^ As the sole renewable resource in nature containing aromatic carbons, lignin boasts abundant functional groups (hydroxyl, methoxy, aldehyde, ether, etc.), commendable biodegradability, and antioxidative properties. Consequently, it emerges as a promising raw material for synthesizing high‐value bio‐based products, offering a potential remedy to mitigate fossil fuel consumption.^[107]^ Presently, lignin finds application in the production of liquid fuels, aromatic chemicals (e.g., benzene, toluene, xylene, benzoic acid, vanillin), as well as simple phenols (e.g., phenol, eugenol, catechol, vanillin), extensively utilized in agriculture, chemical industry, pharmaceuticals, materials, and beyond.^[108]^ For instance, by preserving the macromolecular structure of lignin and leveraging its rich reactive groups as carriers for fertilizers, controlled release of nutrients can be achieved effectively through encapsulation, adsorption, and other techniques while ensuring degradation yields no pollutants. Lignin sulfonates serve as ubiquitous concrete water reducers in the construction sector, with modified variants achieving performance levels akin to widely employed naphthalene‐based superplasticizers. Moreover, these macromolecular structures serve as precursors for synthesizing diverse functional polymers, including polyurethanes, polyesters, epoxy resins among others.

Lignin can undergo depolymerization to yield oligomers and small molecules, which can be further processed and utilized as fuels or chemicals. Effective depolymerization techniques for lignin encompass pyrolysis and liquid‐phase thermoconversion. Liquid‐phase thermoconversion can be subdivided into oxidative depolymerization, reductive depolymerization, acid/base catalyzed depolymerization, and biodepolymerization. The resultant products of lignin depolymerization are bio‐oils, intricate blends comprising compounds with elevated oxygen content, encompassing acids, ketones, alcohols, ethers, esters, aldehydes, phenols, furans, and more. Nevertheless, owing to the intricate nature of bio‐oils, their high oxygen content, and low calorific value, they often fall short of the requisites for high‐grade applications. Enhancing the quality of bio‐oils conventionally involves HDO processes.^[109]^ In practical research, phenolic compounds, ethers, and analogous substances are frequently chosen as platform molecules derived from lignin. Notable lignin‐derived platform molecules include phenol, cresol, anisole, and guaiacol.

#### 4.2.1 Phenol

**FIGURE 10:**
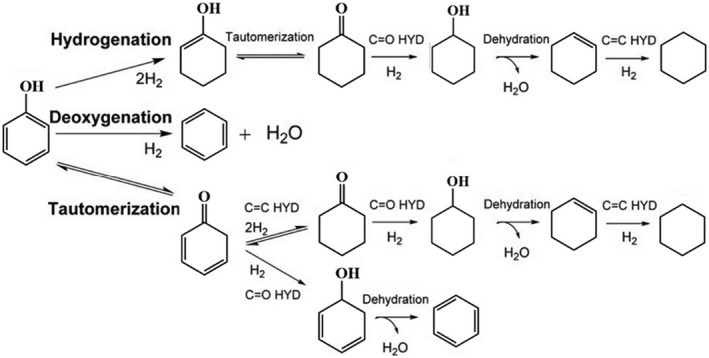
Typical route for the conversion of phenol. Reproduced with permission.^[110]^ Copyright 2016, Wiley Online Library.

Phenol stands out as a high‐value phenolic compound crucial for fuel synthesis, resin production, and fiber manufacturing among other vital applications. The conversion of phenol primarily occurs through three pathways (as depicted in Figure 10 ^[110]^): (1) Hydrogenation of the benzene ring within phenol initiates the formation of cyclohexanone or cyclohexanol, subsequently undergoing dehydration to yield cyclohexene, and finally dehydrogenation to produce cycloalkanes. (2) Direct deoxygenation of phenol results in the formation of benzene. (3) Phenol undergoes intermolecular isomerization, yielding a ketone intermediate (2,4‐cyclohexadienone), which upon hydrogenation generates either benzene or cycloalkanes. Cycloalcohol compounds serve as pivotal intermediates across various chemical processes, finding extensive applications in textiles, pharmaceuticals, cosmetics, and diverse chemical sectors. Notably, cyclohexanol emerges as a fundamental raw material within the polymer industry, acting as a precursor for nylon and various plasticizers. Moreover, its integration as a significant additive in diesel fuel not only reduces particulate matter and nitrogen oxide emissions but also enhances overall fuel performance. Further dehydration and hydrogenation of cyclohexanol yield cyclohexane, which finds utility as both a fuel and solvent. The type of support material influences the reaction pathway of phenol hydrogenation on catalysts. Zhang et al.^[111]^ found that with Ni‐based catalysts supported on SiO_2_, phenol is initially hydrogenated to cyclohexanol, which then undergoes deoxygenation to form cyclohexane. However, when γ‐Al_2_O_3_ is used as the support, Lewis acid sites on the surface preferentially activate the C‐O bond, generating benzene as an intermediate, which then undergoes hydrogenation to produce cyclohexane. Transition metal intermetallic compounds composed of transition metal elements and heteroatoms (N, C, P, O, S, etc.) not only exhibit properties similar to noble metals but also demonstrate superior sulfur resistance, carbon resistance, and anti‐poisoning capabilities. Therefore, they are widely used in lignin hydrogenation reactions.^[112]^ Metals such as Ru and Ni, after phosphorization treatment, reduce the electron density on the metal surface, effectively promoting the adsorption and reaction of benzene rings and oxygen‐containing groups at active sites. Yu et al.^[113]^ prepared a single Ni_3_P catalyst, which showed higher activity than a 1 wt% Pd/SiO_2_ catalyst in catalyzing the hydrogenation of phenol to cyclohexanol.

#### 4.2.2 Cresol

**FIGURE 11:**
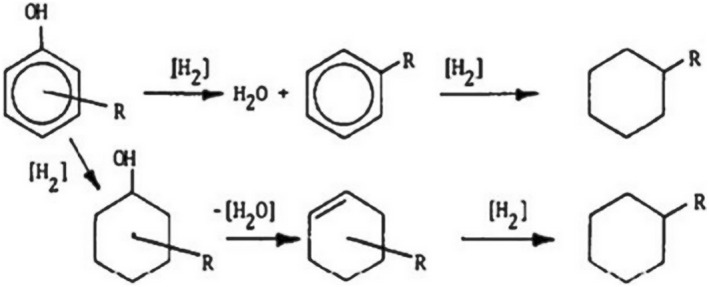
Typical route for the conversion of cresol. Reproduced with permission.^[115]^ Copyright 1983, Elsevier.

Cresol ranks among the most valuable organic fine chemicals, finding application in the synthesis of phenolic resins, antioxidants, insecticides, pesticides, pharmaceuticals, dyes, and various other fields.^[114]^ Cresol manifests in three isomeric forms: ortho‐cresol, meta‐cresol, and para‐cresol, with distinct reactivity patterns among different phenolic compounds following this order: phenol > meta‐cresol > ortho‐cresol > para‐cresol. Two primary conversion pathways for cresol are identified (as depicted in Figure 11 ^[115]^): (1) Direct deoxygenation of cresol leads to the formation of toluene, subsequently undergoing hydrogenation to yield methylcyclohexane. (2) Hydrogenation of cresol initiates the formation of methylcyclohexanol, which upon dehydration yields methylcyclohexene, and finally, hydrogenation produces methylcyclohexane. Methylcyclohexane is characterized by a high octane rating, establishing it as a chemically valuable product with enhanced worth. Through density functional theory calculations, Nørskov et al. found that the binding energy of oxygen atoms to metallic Ru in water vapor (−0.05 eV) is significantly lower than that of Pd and Pt (1.53 and 1.57 eV, respectively), confirming Ru's strong affinity for oxygen.^[116]^ Ma et al.^[117]^ used Ru/Nb_2_O_5_ catalysts for the HDO of p‐cresol in aqueous phase, achieving hydrocarbon yields of up to 99.1% for hydrocarbons, with an 88% selectivity towards aromatics. Transition metal catalysts (Co, Ni, Fe, Cu, etc.) exhibit lower hydrogenation activity compared to noble metal catalysts, but due to their lower cost, they are widely applied in lignin hydrogenation reactions. Yang et al.^[118]^ modified Ni/SiO_2_ catalysts with small amounts of MoO_x_, showing that in the fixed‐bed hydrogenation of m‐cresol, the main product shifted from CH_4_ in Ni/SiO_2_ to toluene in NiMo/SiO_2_.

#### 4.2.3 Anisole

**FIGURE 12:**
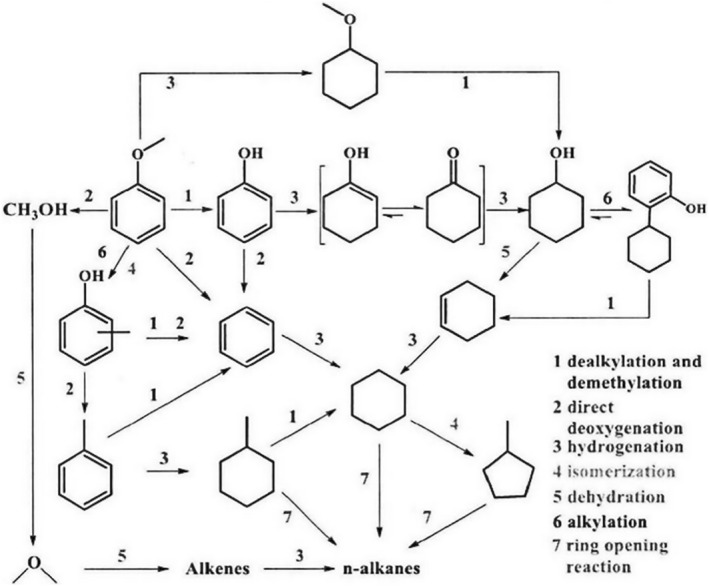
Typical route for the conversion of anisole. Reproduced with permission.^[119]^ Copyright 2015, Elsevier.

Anisole, an aromatic ether, finds diverse applications in resin synthesis, organic solvents, fragrances, insect repellents and so on. Four conversion pathways for anisole are delineated, as depicted in Figure 12 ^[119]^: (1) Initially, anisole undergoes demethylation to yield phenol, subsequently subjected to hydrogenation and deoxygenation, resulting in the formation of benzene or cyclohexane. (2) Direct cleavage of the methoxy group in anisole yields benzene and methanol. (3) Hydrogenation of the aromatic ring in anisole generates methoxycyclohexane, which, upon demethylation, produces cyclohexanol. Dehydration of cyclohexanol yields cyclohexene, followed by hydrogenation to yield cyclohexane. (4) The methyl group in anisole undergoes transfer, yielding cresol, xylenols, etc., which subsequently undergo hydrogenation and deoxygenation to yield cycloalkanes. In the hydrogen deoxygenation of anisole, the synergistic effect between the active metal and the acidic sites of the support has been widely studied.^[120]^ Additionally, the porosity of the catalyst is also a crucial factor in hydrogenation reactions. Li et al.^[121]^ investigated the effect of the porosity of Ni/HZSM‐5 on the conversion of anisole to cyclohexane. They found that with a pore volume of 0.304 cm^3^/g, the conversion rate of anisole was only 3%. However, increasing the pore volume to 0.423 cm^3^/g raised the conversion rate to 94%, demonstrating the significant role of catalyst porosity in enhancing reaction activity in addition to the critical role of acidic sites. Furthermore, incorporating oxygen‐affinic metals into the catalyst can facilitate the direct deoxygenation of anisole via oxygen vacancies. Phan et al.^[122]^ introduced the oxygen‐affinic element Fe into Ru/meso‐TiO_2_ catalysts, achieving an 80% selectivity towards benzene at 250°C, which confirmed that the catalyst activity is related to the number of surface oxygen vacancies on the support. The content and particle size of the loaded metal also affect catalyst performance. Zhao et al.^[123]^ found that Ru/NbOPO_4_ catalysts exhibited the highest activity with a Ru loading of 5 wt%. Beyond this amount, an increase in Ru nanoparticle size led to metal agglomeration and a decrease in active sites, resulting in reduced catalyst activity.

#### 4.2.4 Guaiacol

**FIGURE 13:**
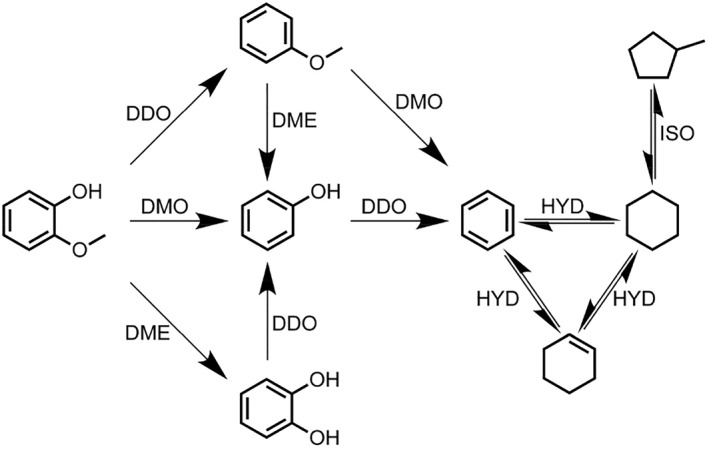
Typical route for the conversion of guaiacol. Reproduced with permission.^[124]^ Copyright 2017, ACS Publications.

Guaiacol finds application in various fragrance productions such as eugenol, vanillin, and synthetic musk. It is also utilized in synthesizing potassium guaiacolsulfonate employed as a local anesthetic or preservative. Additionally, its reducibility renders it suitable for incorporation in cosmetics as an antioxidant. Guaiacol molecules feature three distinct C‐O bonds, leading to three conversion pathways as delineated in Figure 13.^[124]^ In pathway one, guaiacol undergoes demethylation, yielding pyrocatechol and methane, followed by gradual hydroxy group elimination resulting in phenol. Pathway two involves guaiacol's demethoxylation to produce phenol and methanol. Pathway three entails hydroxy group elimination, generating anisole. Hydrogenation and deoxygenation processes yield partially hydrogenated oxygen‐containing compounds such as phenol, catechol, and cyclohexanol as well as fully hydrogenated hydrocarbon compounds. Catechol, among these, serves as a vital precursor in the pesticide, perfume, and pharmaceutical industries. Noble metal catalysts (Pd, Pt, Rh, Ru, etc.) are widely used in hydrogenation refining processes due to their exceptional hydrogenation activity. For example, Wang et al.^[125]^ prepared Ru metal catalysts supported on Y‐type zeolites, which generated products primarily consisting of alkanes during hydrogenation of guaiacol and real lignin. Typically, noble metals preferentially adsorb the benzene rings in lignin model compounds during hydrogenation, leading to deep hydrogenation of the benzene ring to form cycloalkane products. In recent years, researchers have effectively suppressed the hydrogenation capability of active metals by optimizing supports and employing bimetallic strategies, thereby favoring the production of aromatic compounds. Shao et al.^[126]^ compared the activity of Ru metal catalysts supported on different oxides in lignin hydrogenation reactions and found that the Ru/Nb_2_O_5_ catalyst, due to its unique C‐O bond adsorption and activation capabilities, could selectively catalyze the conversion of lignin to aromatic compounds.

## 5 CONCLUSION AND OUTLOOK

With the continuous consumption of fossil fuels, energy shortages and environmental pollution have become pressing issues for human development. Biomass resources, as an emerging renewable resource, can enhance resource utilization and reduce dependence on fossil fuels, showing great development potential. Therefore, effective development and utilization of biomass resources have become a research hotspot worldwide. This paper introduces the structure of biomass and various pretreatment methods, highlighting that different methods can be employed depending on the desired products to achieve green, efficient, and economically viable processing. It provides a solid foundation for the subsequent efficient conversion and utilization of lignocellulose. The paper also summarizes the advantages and disadvantages of different methods, offering new ideas and approaches for the depolymerization of lignocellulose into oligosaccharides or platform molecules. Additionally, it discusses biomass conversion and valorization methods, detailing the value and applications of 12 cellulose/hemicellulose‐based derivatives and 4 lignin‐based derivatives. These derivatives can further be converted into higher‐value chemicals or fuels through reactions such as hydrogenation, dehydration, and reforming, providing theoretical support and clear pathways for the utilization of lignocellulose and laying a strong foundation for industrialization, while also setting higher goals for future development.

However, research on biomass resources is still in the development stage, and there remains a long way to go in converting biomass into high‐value chemicals and fuels. Currently, there are still shortcomings in the depolymerization, separation, and extraction methods of lignocellulose components, such as high chemical consumption, severe pollution, complex procedures, and low yields. Thus, the effective utilization of target components is not yet achievable, requiring more research on the depolymerization mechanisms and processing methods for lignocellulose. Furthermore, the key to lignocellulose conversion lies in the efficient and selective cleavage of C‐O or C‐C bonds and the selective hydrogenation of C=O bonds, which hinders the industrialization of lignocellulose valorization. Solutions involve designing and regulating catalysts to achieve higher activity for reactants and higher selectivity for target products, while combining theoretical calculations to guide catalyst selection, composition, and reaction pathways. Given the issues with existing catalytic conversion systems, such as complex catalyst preparation processes, expensive raw materials, low catalytic efficiency, numerous by‐products, and difficulty in catalyst recovery, developing low‐cost, highly selective, and high‐activity green catalytic materials for biomass platform compound valorization is of significant importance.

## CONFLICT OF INTEREST STATEMENT

The authors declare no conflicts of interest.

## Data Availability

Data sharing is not applicable to this article as no new data were created or analyzed in this study.
